# Strategic analysis of the drought resilience of water supply systems

**DOI:** 10.1098/rsta.2021.0292

**Published:** 2022-12-12

**Authors:** Anna Murgatroyd, Helen Gavin, Olivia Becher, Gemma Coxon, Doug Hunt, Emily Fallon, Jonny Wilson, Gokhan Cuceloglu, Jim W. Hall

**Affiliations:** ^1^ Environmental Change Institute, University of Oxford, Oxford OX1 2JD, UK; ^2^ Ricardo plc, Shoreham-by-Sea, West Sussex BN43 5FG, UK; ^3^ School of Geographical Sciences, University of Bristol, Bristol BS8 1SS, UK; ^4^ DHCR Ltd, Leatherhead KT22 8SQ, UK; ^5^ Environment Agency, Bristol UK

**Keywords:** drought, hydrology, water resources

## Abstract

Severe droughts can result in shortages of water supplies, with widespread social and economic consequences. Here we use a coupled simulation model to assess the reliability of public water supplies in England, in the context of changing scenarios of water demand, water regulation and climate change. The coupled simulation model combines climate simulations, a national-scale hydrological model and a national-scale water resource systems model to demonstrate how extreme meteorological droughts translate into hydrological droughts and water shortages for water users. We use this model to explore the effectiveness of strategic water resource options that are being planned in England to secure water supplies to most of England's population up to a drought return period of 1 in 500 years. We conclude that it is possible to achieve a 1-in-500-years standard in locations where strategic resource options are used, while also reducing water abstraction to restore the aquatic environment. However, the target will be easier to achieve if effective steps are also taken to reduce water demand.

This article is part of the Royal Society Science+ meeting issue ‘Drought risk in the Anthropocene’.

## 1. Introduction

One of the most serious impacts of droughts is upon municipal water supplies. These are the supplies of treated water to homes and businesses that are relied upon for human consumption, hygiene and other essential services worldwide. Because of the societal and economic importance of municipal water supplies, they are usually prioritized at times of droughts and water scarcity, so that available water is, as far as possible, reallocated from other purposes (notably agriculture) to ensure continued supply to urban areas and people [1–3]. However, during the most severe droughts, the water supplies that urban water systems rely upon can become exhausted, which results in water shortages for municipal water users. These shortages may manifest as temporary restrictions on the use of water, or even cuts in piped water supplies with substitution by severely rationed water from other sources (water tankering in high-income countries, bottled water etc.). The economic consequences of these scenarios are potentially devastating [4,5], although some low-income countries have to cope with intermittent municipal water supplies more or less constantly and not just in times of drought [6].

As a result of the major impacts of severe water shortages, water utilities impose less severe restrictions on water use during the onset of droughts in an attempt to reduce demand and conserve water. This may involve media campaigns calling for voluntary adoption of water efficiencies or bans on ‘non-essential’ uses of water, such as watering gardens or car-wash businesses. This creates a dynamic interplay between the onset of drought and adaptive reductions in water demand, which may be sufficient to avert or delay the imposition of more severe restrictions on water use [7].

Dramatic shortages or near-shortages during droughts have occurred in Barcelona (2008), Melbourne (2009) and Cape Town (2018), among other locations. These events, and concerns about water scarcity in other cities, are driven by increasing demand for water (driven in turn by urban population growth), natural climatic variability and climate change, and in some instances inadequate water infrastructure planning and asset management [8–10]. In addition, in many locations, the surface water and groundwater resources that have provided supplies for people and agriculture are over-exploited to an extent that is harming the natural environment [11,12]. The over-exploitation of natural water resources has resulted in some water bodies becoming so polluted (for example due to salinization of over-exploited aquifers or inadequate capacity to dilute and assimilate wastewater discharges) that supplies are unfit for human use [13,14]. Without a doubt, resilient water systems are required to guarantee safe and reliable water supply now and into the future.

The notion of ‘resilience’ of water supplies has been widely examined in the literature. Hashimoto's [15, p. 16] discussion of reliability, resilience and vulnerability of water supplies associated resilience with ‘how quickly a system is likely to recover or bounce back from failure once failure has occurred’. They consider reliability to be the frequency or probability that a system is in a failed state and vulnerability to be the likely magnitude of a failure when it occurs. More generally, resilience is thought of as the ‘ability of a system, community or society exposed to hazards to resist, absorb, accommodate and recover from the effects of a hazard in a timely and efficient manner’ [16]. This definition encompasses the likelihood or frequency of disturbance to a system and the duration before the system has recovered. Thus, metrics of resilience in water supply systems should incorporate quantification of (i) frequency or probability that water shortages of given severity occur, (ii) the duration of those shortages and (iii) the number of people affected by those shortages [17,18]. We will use these three factors to quantify water resource system resilience in this paper. We consider shortages that are deliberately imposed to limit demand in the same way as shortages that are completely unavoidable.

In this paper, we adopt the ‘trilemma’ concept commonly used in the field of energy systems [19] to explore resilient options for water supplies that are provided affordably, reliably and in a way that does not harmfully impact the environment [20]. The ‘water trilemma’ can appear as a constrained optimization problem with two objectives: weighing up security of supply—measured in terms of the expected frequency, duration and severity of water use restrictions—versus cost, subject to environmental constraints. In many instances the environmental objective of the ‘water trilemma’ is encoded as regulatory limits on water withdrawals from the environment, in order to maintain or restore environmental flow requirements in rivers and wetland habitats. The cost of intervening in water supply systems includes the capital cost of developing or expanding water sources, storage, transfer, treatment and distribution infrastructure, as well as the operating costs. It could also include the cost associated with reducing water demand, by paying for water efficiency improvements or fixing leaks, or the regulatory costs associated with drought events. In some countries, these costs are recovered through the payments made by water users, while in others governments may contribute to these costs from general taxation. In any case, there is a tension between enhancing the security and sustainability of water supplies and affordability for water users.

Growing pressures on water supplies mean that new options and operational rules are being widely considered [21–23]. These include actions to provide new water supplies, steps to reduce demand, and measures to restore surface water and groundwater resources to sustainable levels. When locally available resources are scarce, water planners explore water availability over increasingly wide areas. Long-distance water transfers have existed for many years, including transfers that enabled massive urbanization in southern California and the Lesotho Highlands transfer between Lesotho and South Africa. These transfers present water planners with a trilemma between cost, revenue and reliability [24].

Large-scale strategic water projects pose technical challenges in assessing their resilience and raise potential environmental, political and social concerns [25]. Despite this, few planning frameworks exist to support inter- or cross-basin planning decisions. More commonly, research has focussed on building the resilience of water supply infrastructure in independent water systems. For example, in [26] a computational framework for evaluating future policies for the Folsom Reservoir in California is presented, and in [27] a planning problem at a reservoir in Mombasa, Kenya, is overcome. While these approaches provide robust assessments for intra-basin supply solutions, they are limited when used for planning problems which span multiple supply systems.

The construction and management of large-scale supply infrastructure should account for water availability in times of drought, which may necessitate the incorporation of storage reservoirs in the supply system. In particular, inter-region strategic transfers require consideration of the spatial characteristics of future drought hydrology [28]. A large-scale strategic scheme will be more vulnerable to the impacts of drought if droughts are expected to be widespread and spatially correlated across multiple regions, compared with regions that are hydrologically distinct [29,30].

Furthermore, it cannot be assumed that the water supplied by a set of options that are all implemented will be the same as the sum of the results from each of the options implemented individually. Simple water planning methodologies [31] have tended to treat different supply options additively, which is not realistic if there are large-scale system interactions. Testing new supply infrastructure in connected water systems requires an approach that accounts for the spatial characteristics of droughts and dynamic system interactions between the operations of different supply options.

In this paper, we analyse the effects of spatially extensive droughts which span multiple water planning regions and test the potential effectiveness of sets of strategic water resource options, in the context of a range of scenarios for water demand, climate change and environmental regulation. An extensive ensemble of synthetic droughts is used to stress-test different water supply options, individually and in combination. The improvements in water security achieved by the new strategic water supply options are compared with the costs of the options. The methodology is applied to strategic water supply options that are currently being considered by water companies and their regulators in England. The research is novel in its use of synthetic widespread droughts to stress-test a national, complex water supply system, using a simulation model which has been well-calibrated against observed system performance across a large number of reservoirs. It provides a methodology for managing the resilience of municipal water supplies in the face of growing social, environmental and climatic pressures typical of the Anthropocene era.

## 2. England's water resource system

The focus of this study is on the security of water supplies in England. Given that significant sources of water supply in England are in the upper reaches of river catchments in Wales, the analysis also extends into Wales.

Water availability varies significantly across England and Wales. In mountainous areas of Wales rainfall can exceed 3000 mm per year, while in London it averages just below 650 mm per year. For context, average annual rainfall surpasses 1150 mm per year across the UK, 2900 mm in Costa Rica, 840 mm in Ethiopia and 530 mm in Australia. Water resources in the North and West of England are primarily surface water, while there are large aquifers in the South and East of England, where a large proportion of the water supply is abstracted from groundwater.

The water supply system in England is a complex multi-scale network. Public water supply is the water that is taken from rivers, reservoirs and groundwater and supplied through the networks of 20 major water companies to homes and businesses in England. In total, England's water companies currently provide 14 Mm^3^ d^−1^ (million cubic meters per day) to public water supplies. Of this water, more than half is used in households, with the rest being evenly split between water used in non-domestic situations, such as by industrial and business users, and water lost through leaks in water companies' and customers’ pipes.

Some resources (e.g. small boreholes) supply just a few communities, while others supply populations of millions of people and entail major storage and long-distance water transfer. In England, there is currently 2.385 Mm^3^ of reservoir storage across 740 reservoirs. The network is also highly connected: of its 97 river catchments, as defined by the Environment Agency [32], 80 are connected to their neighbours via a river or public water supply transfer (and even more if small transfers less than 2000 m^3^ d^−1^ are considered). The combination of high capacity and high connectivity in a domain that experiences droughts means that the interplay between droughts and water security is a crucial issue for water resource management across the country.

England and Wales have experienced several severe droughts over the past 200 years [33] and are increasingly confronted with conditions experienced in arid and semi-arid regions elsewhere in the world. Both historic and current droughts have had significant impacts, such as failure of crops, environmental degradations, wildfires and restrictions on water use by businesses and householders [34]. The 1975–1976 drought is considered one of England's most severe droughts, with river flow deficits occurring across the county and standpipes implemented in regions experiencing extreme shortages. More recently, prolonged dry conditions in 2022 prompted water rationing in Yorkshire, Oxfordshire, Hampshire, Kent, Sussex and London.

Water resources in England are under growing pressures due to population growth, climate change and the need to restore the aquatic environment [7,35]. Summer droughts are expected to become increasingly common in the future [36]. The Environment Agency [37] estimates that if no action is taken between 2025 and 2050, around 3.435 Mm^3^ d^−1^ of extra capacity is likely to be needed in England by 2050 to meet future pressures on public water supply. While there is growing uncertainty beyond 2050, the Environment Agency estimates that approximately 5.5–6.0 Mm^3^ d^−1^ of additional water may be needed between 2025 and 2100. The need can be met by a combination of increasing supply, moving water within and between river basins (transfers), reducing leakage, and managing demand through improved water efficiency.

To plan for future threats to water supplies, water companies in England are required by law to produce water resource management plans every five years, which set out projections of water demand and options for water supplies to meet demand for the coming 25 years and beyond [35]. The plans are regulated by the Environment Agency and Ofwat (the economic regulator). To reduce the risk of drought impacts, water companies are required to demonstrate their supply reliability under the worst droughts on record and stochastic events, providing an estimate of frequency with which water use restrictions of given severity are expected to be imposed. Restrictions on non-essential uses of water (often referred to as hosepipe bans) constitute a so-called ‘Level 3’ impact, while severe water rationing is denoted ‘Level 4’.

Given the growing challenge to water supplies in some parts of England, there is mounting interest in the use of strategic water transfers to build system resilience. There have been interconnections between some water resource zones (WRZs), and bulk water transfers between water companies, for many years. However, until recently there was no national-scale water resource systems analysis to support national-scale decisions. A first version of such an analysis was led by the water industry body Water UK [4]. The National Infrastructure Commission examined the risk of droughts in their report *Preparing for a drier future* [5], which argued for heightened resilience to extreme droughts up to a 1 : 500 year return period, through a combination of demand reduction, leakage reduction and new strategic infrastructure including storage, transfers, wastewater reuse and desalination. Subsequently, the Environment Agency [37] published national scenarios of options for meeting future water needs. This analysis illustrates a growing commitment to regional and national-scale water resources planning, which is a significant departure from planning arrangements that have been focussed at a water company scale ever since privatization of the water industry in 1989.

Alongside the growing commitment to the resilience of water supplies in extreme droughts, there is widespread recognition of the need to restore and enhance the resilience of the aquatic environment. Aquatic ecosystems are threatened by the interacting threats of over-abstraction and pollution. The Environment Agency estimates that 9% of catchments are unsustainably abstracted while a further 7% ‘may be’ unsustainably abstracted [38]. Only 14% of rivers in England can currently claim to have good ecological status [39]. In view of these threats, the government has a programme of restoring water abstractions to a more sustainable level, by altering abstraction licences that are held by water companies and other abstractors.

The statutory requirement to ensure resilience of water supplies up to a 1 : 500 year drought by 2039 [40] represents a considerable technical challenge. Although Britain benefits from unusually long hydrological records, which have been extended through archival means and the use of proxies [41], analysing a 1 : 500 year return period involves extending well beyond the historic record. Given the impacts of climate change, which may change weather circulation patterns that influence the severity and duration of extreme droughts [42,43], analysis of future droughts at this return period needs to take account of evidence from climate science and climate modelling. In addition, the spatial characteristics of droughts will significantly influence their impacts on water supplies [28,29].

The resilience of water supplies can be assessed using simulation models of water resource systems, which simulate water availability in surface water and groundwater sources, water withdrawals, storage, transfers and water use [25]. Assessing the risk of water shortage in extreme droughts is achieved through testing the water resource system model on a large ensemble of observed and synthetic droughts that represent present and future climate conditions, as well as projections of water demand and scenarios of environmental regulation [44,45]. In this paper, we present and demonstrate such an approach for the assessment of options for strategic water supplies in England.

## 3. Data and modelling set-up

### (a) Overview

**Figure 1.**
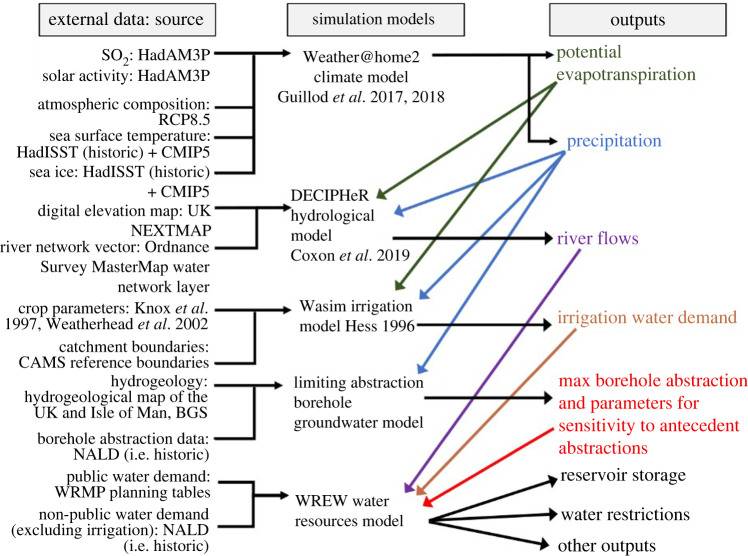
Overview of the workflow, showing the main data sources, models and outputs (after [28]). (Online version in colour.)

A coupled modelling system, the Water Resources England and Wales (WREW) model, has been developed to analyse strategic water resources in the context of large-scale drivers of change, including climate, abstraction reform and changing demand. WREW includes all major water supply infrastructure described in PR19 plans (300 reservoirs, boreholes, transfers, water treatment works, pumped storage, desalination plants and river abstraction points) that are connected into England and Wales's wider water network via any river or transfer of significance (defined following stakeholder engagement as greater than 2000 m^3^ d^−1^). It also includes licence conditions which regulate water abstractions, operational rules, control curves and asset locations where necessary. It is configured to perform simulations on a daily time step. It covers more than 90% of England and Wales's population and public water use, with non-public water users represented at catchment scale. Figure 1 gives an overview of the WREW workflow, showing the main data sources, models and outputs. A full description of the WREW model can be found in [28].

### (b) Climatic inputs

Simulated weather conditions obtained from the Weather@home2 climate modelling exercise were used to create a large ensemble of possible drought conditions, validated based on observations, following a linear bias correction of precipitation estimates [42]. Weather@home2 consists of a global climate model (HadAM3P) with a nested regional climate model (HadRM3P, 25 km grid scale) and driven by sea surface temperatures. Further validation has been conducted to assess the performance in modelling drought extremes [46]. Precipitation and potential evapotranspiration (derived via the Penman–Monteith model with the stomatal resistance adjusted for future time slices) were used in three scenarios as follows:

— 100 realizations of a 30-year (1975–2004) ‘Baseline’ with different initial atmospheric conditions to create the ensemble but each using the same historical sea surface temperature (SST) and sea ice observations from the HadISST dataset [47,48]. This was used for model calibration and validation.
— 100 realizations of a 30-year (2020–2049) ‘Near Future’ ensemble, driven with the 50th-percentile SST and sea ice projections from CMIP5 [49] for the RCP8.5 greenhouse gas concentration scenario [50], which represents the high end of the IPCC's scenarios and hence a reasonable upper bound on climate change. This was used as the ‘Central’ scenario for the planning period.
— 100 realizations of a 30-year (2070–2099) ‘Far Future’ ensemble, which also uses the 50th-percentile SST and sea ice from CMIP5 RCP8.5 emission scenarios. This scenario was used as an additional stress-test to expose the system to more severe climatic conditions.

Though the future scenarios are associated with specific time frames, they are used here as an ensemble of stress-tests, with the Far Future ensemble representing climatically more severe conditions than the Near Future ensemble. In addition, specific severe droughts extending over critical regions and the whole country were extracted from the Weather@home2 ensemble to observe the response of the simulated systems.

### (c) Surface water hydrology

The DECIPHeR hydrological model [51] was used to simulate river flows. The model was calibrated to data from 1366 flow gauges across Great Britain and shown to perform well with respect to four different evaluation metrics covering high flows (Nash–Sutcliffe efficiency), water balance (bias in runoff ratio), low flows (bias in low flow volume) and flow variability (bias in the slope of the flow direction curve) across a wide range of catchments [51].

Ensembles of naturalized flows were generated as input to the water resource simulation model for over 380 input points. The ensemble of naturalized flows for each point consists of flows from the best parameter set, identified either by calibrating the model to daily naturalized flows obtained from the Environment Agency or transferring behavioural parameter sets from donor gauges where no naturalized flows were available. For the model calibration, DECIPHeR was run for a 56-year period (1961–2015) using daily observed precipitation (CEH GEAR [52]) and potential evapotranspiration (CHESS [53]) data as input, and 10 000 parameter sets sampled in a Monte Carlo simulation using wide parameter ranges tested in previous studies [51]. The best parameter set for each point was defined as the top-ranked simulation for Nash–Sutcliffe efficiency and log Nash–Sutcliffe efficiency (using the rank sum) to gain simulations that can capture both high and low flows (particularly important for ensuring correct catchment storages in reservoirs). Thus, for each of the 338 input points, 100 90-year daily flow simulations (created using the best parameter set for each point) were generated from the Weather@home2 projections (covering the Baseline, Near Future and Far Future periods).

### (d) Groundwater

A parametric model was used to estimate groundwater yields, given climatic conditions and antecedent withdrawal. The model was trained using information on groundwater licences within the national abstraction database. This empirical model describes the maximum abstraction rate from a borehole in a given month. This is achieved with a multivariate linear regression between antecedent rainfall, antecedent abstractions and historic abstractions that were made under limiting conditions, for a given borehole of a given geological character. This model is based on the groundwater licence information from the Environment Agency's National Abstraction License Database (NALD), which contains monthly reported public water supply abstraction for 900 boreholes greater than 1000 m^3^ d^−1^.

The empirical groundwater model was used to generate groundwater yields for the majority of water supply boreholes in England and Wales. However, significant discrepancies of outputs between WREW's empirical model and Affinity Water's lumped parameter model were identified during validation and calibration. To improve the accuracy of Affinity Water's groundwater representation in WREW, the Weather@home2 climate data were run through the company's lumped parameter model and the outputs used as groundwater inputs in WREW.

### (e) Water demand

The WREW model (see below) covers more than 90% of England and Wales's population, representing the demand of public and non-public water users. Daily public water demand is calculated at WRZ scale, using estimates of annual demand submitted by water companies to the Environment Agency. The submitted estimates use projections for per capita consumption (PCC), leakage and population to estimate the expected yearly average water use per WRZ. In WREW, an individual WRZ is typically represented by one demand node, although WRZs may be aggregated or disaggregated depending on system connectivity.

Annual demand estimates for each demand scenario are converted to daily demand using an annual profile of demand specific to each WRZ or water company. If an annual profile was not available, a default annual profile was used, taken as the average across all available annual profiles.

The Environment Agency's NALD was used to derive water use for non-public water supply for the years 1999 to 2015, including agriculture and industry. Although less than 1% of abstracted surface water in England is used for irrigated agriculture, this usage is seasonal, highly spatially concentrated and a high-value use, so a seasonal model of agricultural water demand was employed [54].

Empirical data or standard assumptions based on information obtained from water companies have been used to derive non-consumed water volumes and/or effluent, returning it to the model downstream of the abstraction point. A complete description of non-public water demand modelling for WREW is outlined in [28].

### (f) Water resource system model

The water resource system simulation was implemented at a daily time step using the WATHNET software [55]. WATHNET solves a mass balance optimization problem that allocates water between model nodes, via arcs, under both constraints inherent to mass balance (e.g. non-zero flows and storages) and constraints set out by the water system's formulation (e.g. pipe capacities and minimum required river flows).

In order to build a computationally efficient model, a number of assumptions have been made:

— occasional aggregation of multiple reservoirs that supply single treatment works;
— the omission or aggregation of small sources (the lowest non-zero flow is 1000 m^3^ day^−1^);
— representation of water redistribution in unmodelled areas by allowing multiple sources/transfers to deliver water to the same demand node;
— instantaneous flow travel time along arcs (except for aqueducts with known travel times);
— zero evaporation for reservoirs (except where relationships have been provided);
— assumed acceptability of water quality and exclusion of reservoir dead water storage volumes (unless known).

Model calibration was carried out through close interaction with the Environment Agency and water companies over a period of several years. The model has been compared against major reservoir levels, flows and transfer volumes, and the simulation results have been found to be comparable to water company model outputs, with the lowest Nash–Sutcliffe efficiency being 0.7 [28]. Further evidence of model validation in droughts at key reservoirs in England and Wales is given in [46] and compared with regional modelling outputs.

### (g) Scenarios

The strategic options were tested with respect to three scenario dimensions: (i) future water demand, (ii) environmental regulation of water abstractions and (iii) climate change. The Central scenario sought to align with water company regional plans, while the Sensitivity scenarios explored plausible variants on these plans, with a focus on conditions that would increase the risk of water shortages in the future (table 1).

**Table 1.** Summary of scenarios.

			Sensitivity scenario 1: less	Sensitivity scenario 2: high	Sensitivity scenario 3: far future
	Baseline scenario	Central scenario	effective demand reduction	environmental destination	climate change
water demand	PCC from 2021 levels	PCC reduces from current levels to 110–120 l/h/d by 2050; 50% reduction in leakage from 2017/2018 levels	only 50% of PPC reduction in the Central scenario is achieved; 25% reduction in leakage from 2017/2018 levels	PCC reduces from current levels to 110–120 l/h/d by 2050; 50% reduction in leakage from 2017/2018 levels	PCC reduces from current levels to 110–120 l/h/d by 2050; 50% reduction in leakage from 2017/2018 levels
environmental regulation	current regulatory arrangement	current regulatory arrangement plus further measures to recover flows in protected areas	current regulatory arrangement plus further measures to recover flows in protected areas	greater environmental protection for protected areas, SSSI rivers and wetlands, principal salmon and chalk rivers	current regulatory arrangement plus further measures to recover flows in protected areas
climate change	Weather@home2 ensemble for 1975–2004	Weather@home2 ensemble for 2020–2049	Weather@home2 ensemble for 2020–2049	Weather@home2 ensemble for 2020–2049	Weather@home2 ensemble for 2070–2099

### (h) Strategic water resource options

**Figure 2.**
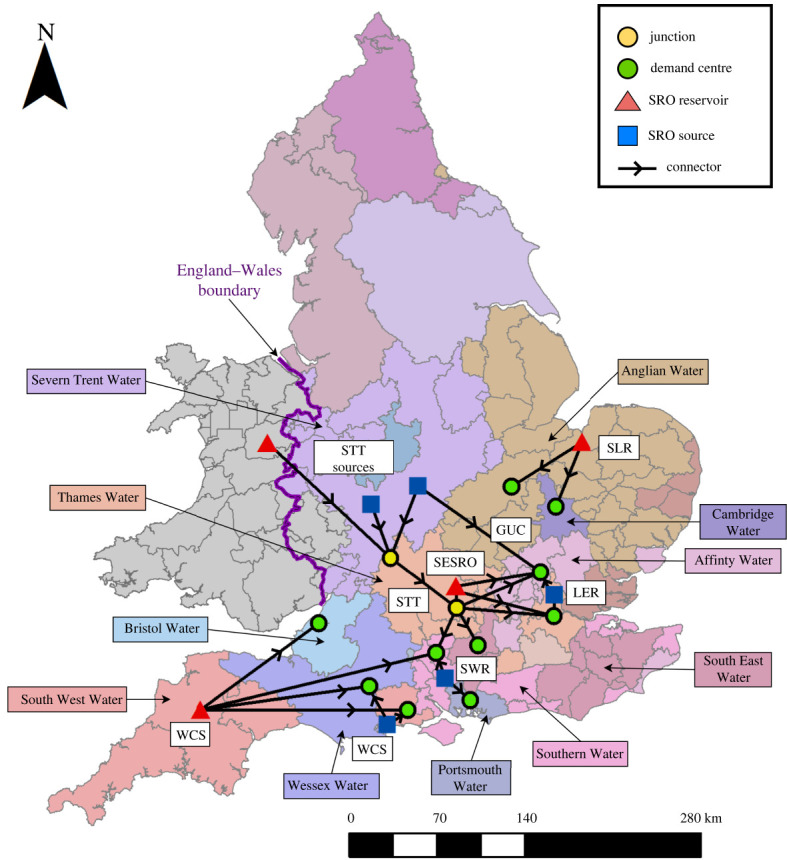
RAPID Gate 1 SROs modelled in WREW and key water companies' locations. WRZs not modelled in WREW are shaded grey. (Online version in colour.)

The strategic resource options (SROs) tested are shown in table 2 and figure 2, including design configurations that incorporate different sub-options, many of which offer a range of capacities or yields for the solutions. We have chosen to test each solution at its maximum capacity or yield, unless dialogue with water companies suggested otherwise. The SROs include major water transfers (STT and GUC), storage reservoirs (SESRO and SLR), water sources (LER, SWR and WCS) and combinations thereof. It should be noted that the SROs and local supply options being planned by water companies through to 2025 are the only new supply options implemented in the model, so other interventions (or supporting options) that water companies are planning beyond 2025 have not been tested.

**Table 2.** SROs tested.

SRO name	type	sub-options(s)	max supply yield (Mm^3^ d^−1^)	recipient	recipient max yield (Mm^3^ d^−1^)
Severn Thames Transfer (STT)	water transfer (river/pipeline)	unsupported component; Vyrnwy component; Minworth component; Mythe component; Netheridge component	0.500	Affinity Water^a^	0.100
				Southern Water^b^	0.080
				South East Water^b^	0.020
				Thames Water	0.500
South East Strategic Reservoir Option (SESRO)	reservoir and water transfer (river)	—	0.321	Affinity Water^a^	0.100
				Southern Water^b^	0.080
				South East Water^b^	0.020
				Thames Water	0.321
London Effluent Reuse (LER)	effluent reuse	Beckton Reuse; Teddington DRA; Mogden Effluent; Mogden South Sewer Scheme	0.350	Affinity Water^a^	0.100
				Thames Water	0.350
Grand Union Canal (GUC)	water transfer (canal)	—	0.100	Affinity Water	0.100
South Lincolnshire and Fenland Reservoirs network (SLR)	reservoir and water transfer (pipeline)	South Lincolnshire Reservoir; Fenland Reservoir	0.220	Anglian Water	0.180
				Cambridge Water	0.040
Southern Water Recycling (SWR)	reservoir, water recycling and transfer (pipeline)	Havant Thicket; Cheddar 2 Reservoir; Poole effluent reuse; Mendips Quarry	0.165	Southern Water	0.140
				Portsmouth	0.025
West Country Sources (WCS)	reservoir, effluent reuse and water transfer (pipeline)	reservoir, effluent reuse and water transfer (pipeline)	0.180	Wessex Water	0.150
				Bristol Water	0.015
				Southern Water	0.030
				South West Water	0.030

^a^Part of the Thames to Affinity Transfer (T2AT) SRO.

^b^Part of the Thames to Southern Transfer (T2ST) SRO.

## 3. Results

### (a) Reference scenarios (without SROs)

**Figure 3.**
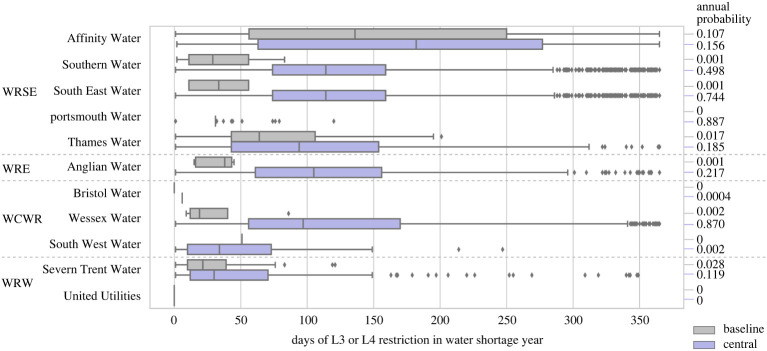
Water shortage event (L3 or L4 restriction) probabilities and durations for the Baseline and Central scenarios. Regions: WRSE = Water Resources South East; WRE = Water Resources East; WCWR = West Country Water Resources; WRW = Water Resources West. Water Resources North (WRN) was omitted as the SROs tested in this paper do not connect to the region. (Online version in colour.)

In all of the scenarios modelled, water shortage events were identified as when Level 3 (L3) or Level 4 (L4) restrictions had to be imposed on water use in WRZs, based on the encoded operational rules. The probability of an annual water restriction is calculated as the proportion of years in the scenario in which restrictions are imposed. Figure 3 shows the simulated probabilities and durations of Level 3 or 4 shortages in the Baseline and Central scenarios for different water companies in England (grouped by water planning regions) if no SROs are constructed.

**Figure 4.**
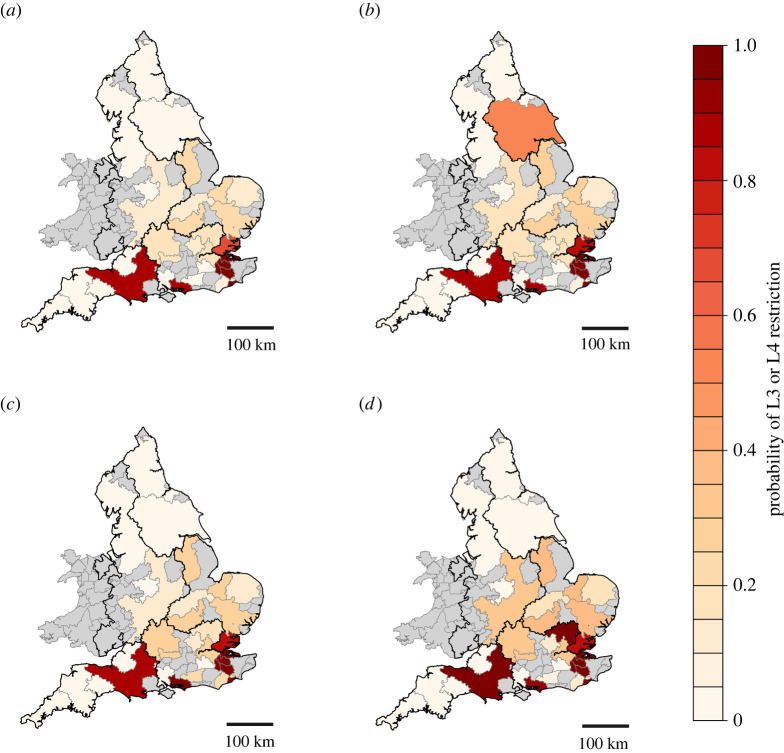
Probability of L3 and L4 restrictions on water use for the Central scenario and three Sensitivity scenarios shown in table 2. WRZs with no estimate of water restrictions are shaded grey; water planning regions are delineated by thick black lines. (*a*) Central scenario; (*b*) high environmental destination Sensitivity scenario; (*c*) less effective demand reduction Sensitivity scenario; and (*d*) far future climate change Sensitivity scenario. (Online version in colour.)

Figure 4 demonstrates that the greatest risks of droughts on water supplies are projected to be in the East and South of England. The probability of water restrictions in the London region is relatively high (0.185) for the Central scenario, caused by a combination of the very large demand in London and relatively limited reservoir storage volume. This means that supplies during droughts are sensitive to variations in low flows, which in turn vary significantly according to the severity of the drought event. The darkest red regions on the map (figure 4) (e.g. Wessex Water, South East Water) are regions with a fine balance between supply and demand.

The high environmental destination scenario (figure 4*b*) results in an increase in the probability of water restrictions for all WRZs relative to the Central scenario where substantial decreases in permitted water abstractions were tested. However, as the South and the East of England contained substantial reductions in permitted water abstractions even in the Central scenario, there is little increase in the probability of water restrictions occurring under the high environmental destination scenario.

If water demand reduction measures fail to meet their 2050 targets by 50% (figure 4*c*), the probability of water restrictions would increase in the South East. This region includes some of the most water-stressed areas in England, where supply only marginally exceeds demand, and therefore failure to achieve targeted demand reductions can be expected to increase the probability of water restrictions. In South East Water's WRZ3, for example, the probability of restriction increases from 0.076 in the Central scenario to 0.245 in the less effective demand reduction scenario.

In the more severe ‘Far Future’ climate change scenario (figure 4*d*), the likelihood of water restrictions is projected to worsen. As the spatial characteristics of shortages projected in the Central and Far Future scenarios are similar, on average, this indicates that the spatial pattern of drought across the country may stay the same in the future, while drought severity will grow (as indicated by the comparatively darker shading).

**Figure 5.**
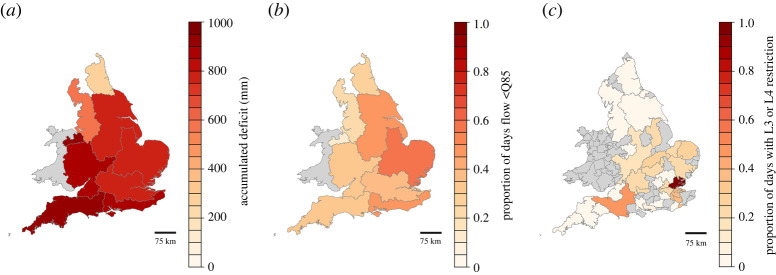
Illustration of a 24-month national meteorological drought event and the associated hydrological and water system response. (*a*) Accumulated deficit (in mm) of drought events below Q50 threshold of effective precipitation for river basins in England (see appendix A in electronic supplementary material for method). (*b*) Proportion of days below Q85 threshold in key rivers in basins in England (see appendix B in electronic supplementary material for method and river locations). (*c*) Proportion of days in WRZ with Level 3 or 4 water use restriction. WRZs with no estimate of water restrictions are shaded grey. (Online version in colour.)

Figure 5 illustrates (i) an extreme 24-month meteorological drought extracted from the Weather@home2 Near Future ensemble, (ii) the corresponding hydrological conditions, using an indicator of low flows, and (iii) the resulting restrictions on public water supplies, measured as the proportion of days in the drought event with a L3 or L4 restriction. The figure suggests that water systems in the South and East of England are most vulnerable to meteorological drought events, with rivers in the Anglian, Thames and Southern river basin regions showing the greatest response to changes in precipitation with the highest frequency of low-flow days in the drought period (i.e. when flows are lower than the 15th percentile of baseline flows: Q85).

Hydrological conditions in the West and North West are less impacted by the national drought event, which is reflected by the lower proportion of low-flow days and days with L3 or L4 restrictions. These regions have a greater quantity of stored water in reservoirs compared to the South and East of England, which provide a buffer in extended periods of low rainfall and when water demand is high.

The drought analysis indicates that the configuration of water infrastructure can be as important as climatological and hydrological factors in the resilience of water supplies.

### (b) Impact of SROs

The effects of SROs, individually and in selected combinations, on the probability of L3 and L4 water restrictions in the Central scenario are given in table 3. Visualization of the results presented in table 3 can be found in appendix C of the electronic supplementary material. In general, reuse schemes show the greatest resilience to future changes in climate and demand, followed by reservoirs and finally transfers. For the most densely populated London WRZ, the inter-basin Severn Thames Transfer, the intra-basin London Effluent Reuse and the South East Strategic Reservoir are the most effective options for reducing the probability of restrictions. SESRO is the most cost-effective option of the three SROs, with the lowest cost per person benefitting from the SRO. The cost–benefit estimates in the table give an indication of the trade-off between water resource system reliability and cost, which is ultimately paid by water users.

**Table 3.** Impact of SROs on probability of water shortages (Central scenario). Water companies: TW** = **Thames Water; AfW** = **Affinity Water; AnW **= **Anglian Water; SW **= **Southern Water.

SRO	primary recipient WRZ(s)	population served (est. 2050)	max estimated SRO present value cost^a^ (£millions)	annual probability of L3/L4 restriction for served population without SRO	annual probability of L3/L4 restriction for served population with SRO	cost per person benefitting from SRO^b^ (£)	annual probability of L3/L4 restriction for served population with all SROs
STT + sources	TW London & SWOX	10 897 787	2459.7	0.1849	0.1112	3063	0.0132
SESRO	TW London & SWOX	10 897 787	1437.7	0.1849	0.1088	1734	0.0132
LER	TW London & SWOX	10 897 787	2381.7	0.1849	0.0736	1964	0.0132
GUC	AfW WRZ 1, 2 & 4	2 585 634	1160	1.0000^c^	0.4864^c^	874	0.0004^c^
SLR	AnW Ruthamford North	1 351 598	3285.7	0.1084	0.0757	74 342	0.0649
	AnW Ruthamford South	1 484 138		0.2436	0.0447	11 131	0.0203
SWR	SW South Hants	852 318	Gate 1 cost not available	0.0190	0.0190	—	0.0190
	SW North Sussex	362 875		0.0401	0.0112	—	0.0070
	Portsmouth Water	907 010		0.8871	0.8879	—	0.8809
WCS	Wessex Water Supply Area	1 565 626	1244	0.8701	0.8205	16 020	0.8209
STT + SESRO	TW London & SWOX	10 897 787	3897.4	0.1849	0.0447	2551	0.0132
GUC + STT	AfW WRZ 1, 2 & 4	2 585 634	3619.7	1.0000^c^	0.0087^c^	141	0.0004^c^
	TW London & SWOX	1 089 7787		0.1849	0.0835	3276	0.0132

^a^SRO costs from RAPID Standard Gate One Key Themes and Assessment Overview. Costs represent the maximum estimated cost of total planning period's indicative solution cost net present value for each of the options with a solution reported for maximum utilization.

^b^Cost per person benefitting from SRO = SRO Present Value Cost (£)/[(Population × L3 or L4 probability without SRO)−(Population ×L3 or L4 probability with SRO)].

^c^Probability of annual supply shortfall used instead of annual probability of L3/L4 restriction.

**Figure 6.**
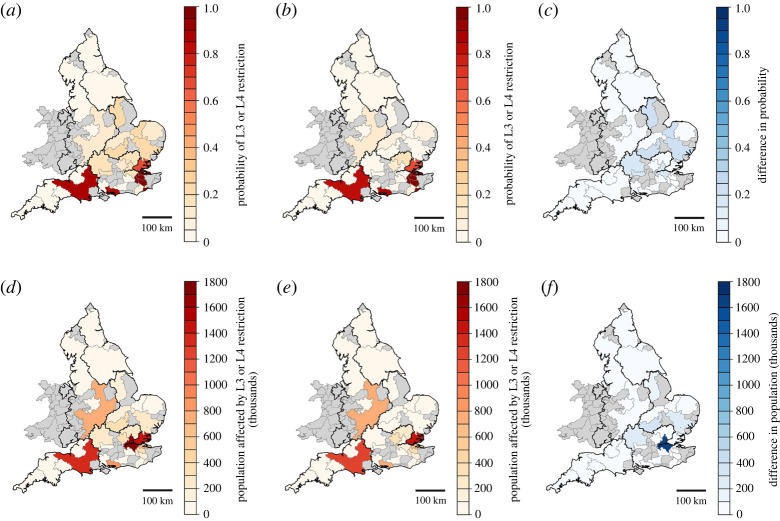
Probability of L3 and L4 water restrictions in the Central scenario without (*a*) and with (*b*) all SROs, and the difference between these probabilities (*c*). Portion of the WRZ population affected by L3 and L4 water restrictions (population × probability) without (*d*) and with (*e*) all SROs, and the difference between these populations (*f*). WRZs with no estimate of water restrictions are shaded grey. (Online version in colour.)

When all of the SROs were implemented in WREW, there was a substantial reduction in both the probability of water restrictions (figure 6*a–c*) and the total population affected by restrictions (figure 6*d–f*) in key water-stressed urban areas in the South and East. The greatest benefit of the SROs, in terms of population affected by restrictions, is observed in the London WRZ. The annual probability of severe water restrictions in London is projected to decrease from 0.185 to 0.0132 in the Central scenario, while the annual probability of water supply shortfalls (i.e. when water demand exceeds the water available for supply) in the same zone is projected to decrease from 0.041 to 0.001 with the addition of SROs, achieving the National Infrastructure Commission's target standard of 0.002.

## 4. Conclusion

A coupled modelling system, WREW, for analysis of drought risk to public water supplies has been established for almost all of England and Wales. The system combines a large ensemble of synthetic droughts generated from climate modelling with national-scale hydrological modelling and a water resource simulation model which covers 90% of the water supply infrastructure in England and Wales. Demand for municipal water supplies is represented and projected into the future using scenarios of population growth and per capita water demand. The model has been calibrated against observed meteorology, river flows and reservoir levels. While it does not represent all of the details of local water supply systems, it provides a strategic overview and is sufficiently resolved to represent the large-scale dynamics of droughts and water resource system response. The WREW modelling system has been used to stress-test the current water resource system and future infrastructure plans under a range of different scenarios of climate change, demand and environmental regulation. This approach of stress-testing large-scale water resource systems with a large ensemble of synthetic drought events represents a transferable methodology for analysis of drought risk in the Anthropocene.

The simulations illustrate how meteorological droughts translate into low water availability and ultimately into water use restrictions and shortages for water users. The patterns of meteorological drought, measured with accumulated deficit of effective precipitation, can translate into different patterns of flows and water shortages depending on the catchment hydrology, infrastructure system and societal response. The results suggest that well-known drought indicators risk showing little resemblance to the ultimate outcomes of droughts for people [56].

The study shows that for locations where data on infrastructure, climate and water demand are available, models such as WREW can be highly valuable in assessing future water security risks. In data-scarce areas, the proposed approach may require pre-feasibility checks and data collection to inform the construction of the coupled modelling system. Inadequate data quality and quantity, in addition to limited understanding of the planning region, may lead to poor parametrization, calibration and validation of the modelling framework. This will increase the likelihood of modelling uncertainties and thus limit the reliability of the coupled modelling approach described here. Advances in open source databases such as OpenStreetMap may make the coupled modelling approaches more accessible for data-scarce regions.

The results for England illustrate that in the absence of further interventions to reduce water demand and provide additional water supplies, there is a high risk of water shortages, in particular in the South East of England. However, active steps are being taken to manage these risks through enhancements to water supplies at the scale of individual water companies, as well as by planning new strategic water resource options that have been analysed in this study. The analysis indicates how SROs interact with each other over a large scale. Their performance depends on the spatial characteristics of the droughts which they are subjected to. Thus, it is important to use large-scale simulation models like WREW in order to properly appraise combinations of SROs which interact in complex ways with each other.

The risk of water shortages in England is anticipated to worsen in scenarios of more severe climate change. In many parts of the country, the need to reduce water withdrawals, in order to protect and restore the aquatic environment, is limiting the water available for human use. This will increase the risk of water shortages, unless further steps are taken to reduce demand and provide additional sustainable supplies. A set of SROs were tested in the WREW model, demonstrating that if these SROs are implemented in combination along with demand management measures, they will be sufficient to achieve the target of resilience to a 1-in-500-year drought in almost all of England. The WREW modelling framework has enabled the creation of a national picture of drought risk in England and Wales and a tool for strategic management of drought risk into the future.

## Data Availability

The Weather@home2 data are available at https://hess.copernicus.org/articles/22/611/2018/. The DECIPHeR model is available from https://gmd.copernicus.org/articles/12/2285/2019/. The WREW model cannot be made available because of commercial limitations by English water companies. Additional data and results are provided in the electronic supplementary material [57].
